# Drought Stress Causes Specific Changes to the Spliceosome and Stress Granule Components

**DOI:** 10.3389/fmolb.2019.00163

**Published:** 2020-01-21

**Authors:** Claudius Marondedze, Ludivine Thomas, Kathryn S. Lilley, Chris Gehring

**Affiliations:** ^1^Department of Biochemistry, Cambridge Centre for Proteomics and Cambridge Systems Biology Centre, University of Cambridge, Cambridge, United Kingdom; ^2^Division of Biological and Environmental Sciences and Engineering, King Abdullah University of Science and Technology, Thuwal, Saudi Arabia; ^3^Department of Chemistry, Biology and Biotechnology, University of Perugia, Perugia, Italy

**Keywords:** RNA-Binding proteins, drought stress, spliceosome, stress granules, mRNA interactome capture, systems analysis

## Abstract

The spliceosome processes RNAs from a pre-RNA state to a mature mRNA thereby influencing RNA availability for translation, localization, and turnover. It consists of complex structures containing RNA-binding proteins (RBPs) essential for post-transcriptional gene expression control. Here we investigate the dynamic modifications of spliceosomal RBPs under stress and in particular drought stress. We do so by mRNA interactome capture in *Arabidopsis thaliana* using label free quantitation. This approach identified 44 proteins associated with the spliceosome and further 32 proteins associated with stress granules. We noted a high enrichment in the motifs RDRR and RSRSRS that are characteristic of RNA interacting proteins. Identification of splicing factors reflect direct and/or indirect stress induced splicing events that have a direct effect on transcriptome and proteome changes under stress. Furthermore, detection of stress granule components is consistent with transcriptional arrest. Identification of drought induced stress granule components is critical in determining common abiotic stress-induced foci that can have biotechnological applications. This study may therefore open ways to modify plant stress responses at a systems level through the modification of key spliceosome components.

## Introduction

Transcription and post-transcriptional gene regulation (PTGR) are the first and main targets in gene expression control. The PTGR regulatory networks involve RNA processing, modification, stabilization, storage, localization, translation, and degradation (Cole, 2001; Lorkovic and Barta, 2002). These processes are tightly controlled, in part by RNA-binding proteins (RBPs). RNA processing and modifications are chiefly carried out by splicing factors, a group of RBPs, constituting the spliceosome complexes. The spliceosome is a large ribonucleoprotein (RNP) complex whose assembly at each intron involves small nuclear RNAs and hundreds of proteins. In eukaryotes, the spliceosome performs the essential processing of RNA; removing introns from pre-mRNA to form a mature mRNA. The highly dynamic composition of the spliceosome and RNPs, in general, orchestrates changes of the transcriptome during growth, development and in response to exogenous cues (Köster et al., 2017). Such consequences point to critical functions of RBPs in controlling the cellular mRNA population at any given time.

It has also been reported that transcriptional arrest leading to the induction of stress granule (SG) formation in response to low oxygen, oxidative, and heat stresses is mediated by RBPs (Anderson and Kedersha, 2006; Ivanov and Nadezhdina, 2006; Weber et al., 2008). SGs are cytoplasmic foci formed from aggregates of non-translated messenger RNPs. It has been proposed that SGs serve as sorting sites, where mRNAs are directed for storage, re-initiation or degradation through transfer to the processing bodies (Kedersha et al., 2005). In animal systems, SGs have been noted to contain poly-adenylated mRNPs in the form of stalled 48S pre-initiation complexes that also contain small ribosomal proteins, translation initiation factors, and RBPs such as poly(A) binding protein (PAB) 1 (Kedersha and Anderson, 2002; Kimball et al., 2003). In plants, only a few proteins have been identified as constituents of the SGs and including the initiation factors eIF4E, RBP47, and UBP1 (Weber et al., 2008) and LaRP1a (Merret et al., 2013). However, a detailed composition of SGs is yet to be fully characterized in plant and other systems under various stresses.

Genome-wide and systems level identification of proteins binding, *in vivo* and in a time- and stimulus- specific manner, to mRNA has been made possible through the use of an interactome capture technology. This method has been applied to obtain the first genome-wide mRNA interactomes in various organisms including human cell lines (Baltz et al., 2012; Castello et al., 2013; Kwon et al., 2013), yeast (*Saccharomyces cerevisiae*) (Beckmann et al., 2015), drosophila (*Drosophila melanogaster*) (Sysoev et al., 2016), macrophages (Liepelt et al., 2016), and higher plants (Marondedze et al., 2016b). Recently, we have shown that an environmental cue such as drought stress, induced by polyethylene glycol (PEG), causes modification of the mRNA interactome affecting potential dual function proteins such as proteins involved in intermediary metabolism (Marondedze et al., 2019). Thus, far, interrogating the mRNA-binding proteome (RB-proteome) has given new insights into the mechanisms underpinning developmental and physiological states of cellular systems. However, the role of the spliceosome in stress-dependent transcriptional changes has remained unclear. Here, were therefore carried out a study to determine if the components of the spliceosome are altered during stress by monitoring changes in the RBPs during drought stress using *Arabidopsis thaliana* as model system. Additionally, we further interrogated the composition of drought induced SGs.

## Methods

### Cell Culture and Treatment

Cells derived from roots of *Arabidopsis thaliana* (ecotype Columbia-0) were grown in liquid medium, as previously described (Marondedze et al., 2013, 2014; Ordonez et al., 2014). The cell cultures used in this study were obtained from Mrs Xiaolan Yu in the Department of Biochemistry at the University of Cambridge. Cells were treated with 40% (v/v) polyethylene glycol (PEG) 6000, a dehydration-inducing agent to mimic drought stress or with equal volumes of media as a negative control. Three biological replicates of cells treated with PEG or mock-treated cells were collected at 1 and 4 h post-treatment. Each time-point treatment had a corresponding mock treatment per replicate. The medium was drained using Stericup® filter unit (Millipore, Billerica, MA), and cells were rinsed with 1X phosphate buffered saline immediately before UV-crosslinking (Marondedze et al., 2016b).

### Abscisic Acid (ABA) Assay

Three biological replicates of cell suspension cultures for each time-point (controls at 0, 1, and 4 h, and 40% PEG treated samples at 1 and 4 h) were subjected to Phytodetek® ABA Immunoassay (Agdia Inc., Elkhart, Indiana, USA) following the manufacturer's instructions. ABA levels were measured and statistically evaluated between each control and treatment time-point. The data for this assay has been published (Marondedze et al., 2019).

### UV-Crosslinking and Interactome Capture

*In vivo* UV-crosslinking and isolation of Arabidopsis RBPs was performed, as previously described (Marondedze et al., 2016b), using a protocol that utilizes a modified method originally optimized for HeLa cells (Castello et al., 2013). Sample from each time-point were split into two, one set for UV-crosslinking and the second set for non UV-crosslinking. Samples for UV-crosslinking were irradiated *in vivo* with UV (254 nm) using a Stratalinker® UV crosslinker (Stratagene, La Jolla, CA) and the mRNA-protein complexes were pulled down using oligo(dT) beads. Purified proteins were analyzed by label free tandem mass spectrometry. Similarly to (Marondedze et al., 2016b), the quality of the mRNA-protein crosslinked complex pull-down was assessed by performing an additional control whereby the sample was treated with RNase T1/A mix (Thermo-Fisher Scientific) and the reaction was performed according to the manufacturer's recommendations. To isolate RBPs, mRNA-protein samples were treated with RNase A/T1 mix to release them from the captured RNA molecules. Crosslinking and isolation of RBPs were evaluated by western blotting using antibodies against polypyrimidine tract-binding protein 1, β-actin (Sigma Aldrich, St Louis, MO, USA) and Histone 3 (Abcam, Cambridge, UK) following the manufacturer's recommendations (see Marondedze et al., 2016b).

### Protein Digestion and Mass Spectrometry

Protein samples were reduced, alkylated, buffer exchanged and digested, as described elsewhere (Marondedze et al., 2016b). Dried peptides were resuspended in 20 μL of 5% (v/v) acetonitrile and 0.1% (v/v) formic acid and analyzed with Q-Exactive™ Hybrid Quadrupole-Orbitrap™ using nano-electrospray ionization (Thermo-Fisher Scientific, San Jose, CA) coupled with a nano-Liquid Chromatography (LC) Dionex Ultimate 3000 Ultra High Performance Liquid Chromatography (UHPLC) (Thermo-Fisher Scientific). Mass spectrometry parameters and run analysis were performed following the protocol described in Marondedze et al. (2016a).

### Mass Spectrometry Data Analysis

Raw files were processed using the Proteome Discoverer v2.1 (Thermo-Fisher Scientific) interlinked with the local MASCOT server (Matrix Science, London, UK). MASCOT searches were carried out against *Arabidopsis thaliana* database [built using the Arabidopsis information resource (TAIR; release 10)] using a precursor mass tolerance of 20 ppm, a fragment ion mass tolerance of ±0.5 Da and strict trypsin specificity allowing up to two missed cleavages, peptide charges of +2, +3, and +4. Carbamidomethyl modification on cysteine residues was used as a fixed modification, oxidation on methionine residues as variable modifications and the decoy database was selected. Further stringency was applied on the peptide spectrum matches (PSMs) by allowing “forward” and “decoy” searches by MASCOT to be re-scored using the Percolator algorithm in Proteome Discoverer v2.1 thus yielding a robust false discovery rate (FDR) of <1%. A minimum of two high confidence peptides per protein was prerequisite for identification using Proteome Discoverer.

### UV-Crosslink Enrichment

Protein enrichment upon UV-crosslinking was performed as previously described (Marondedze et al., 2016b). Proteins that were detected in both the UV-crosslinked samples and the control (non-UV crosslinked samples) were quantitatively analyzed to assess UV-crosslinking enrichment. Normalized intensities of UV-crosslinked samples were quantitatively compared with the normalized intensities of the control (non-UV crosslinked samples), and a log_2_-fold change of ≥2 and *p*-value of ≤0.05 (using Student's T-test corrected for multiple testing using the method of Benjamini and Hochberg (Benjamini and Hochberg, 1995) were applied for proteins to be categorized as enriched RBPs and to be considered for further data analysis.

### Drought Stress Responsive RB-Proteome Analysis

After normalization of the data and UV-crosslink enrichment analysis, proteins from the UV-crosslink enrichment and those that were only identified in the UV-crosslinked samples were used for quantitative analysis. Only proteins detected in at least two biological replicates were included. In this analysis, samples collected at 1 h time point, that is 1 h PEG treated samples and mock treated controls were compared against each other and similarly for the samples collected at 4 h time point. Proteins with a log_2_-fold change ≥1.5 and *p-*value ≤ 0.05 that was corrected for multiple hypotheses testing using the method of Benjamini and Hochberg (Benjamini and Hochberg, 1995) were classified as significantly differentially regulated proteins. Furthermore, to determine the interaction capacity relative to the total soluble protein, a heatmap representing protein abundances were z-score normalized within the total and the RNA-binding protein UV crosslinked samples separately. Hierarchical clustering was performed with the heatmapper (www2.heatmapper.ca) using Spearman Rank Correlation as the distance metric and complete linkage.

### Bioinformatics Analyses

#### Gene Ontology Analyses and Classification

To identify proteins that are components of the spliceosome complex data mining was done using gene ontology (GO) enrichment too AGRIGO (http://www.heatmapper.ca) and pathway analysis using the KEGG mapper (http://www.kegg.jp/kegg/tool/annotate_sequence.html; February 2017), which annotates sequences by BlastKOALA. BlastKOALA is an internal annotation tool in KEGG that assigns KEGG Orthology numbers by BLAST searches against a non-redundant set of KEGG GENES using SSEARCH computation (Kanehisa et al., 2016). Stress granules were determined from previously identified and characterized data in literature (Buchan and Parker, 2009; Chantarachot and Bailey-Serres, 2018; Kosmacz et al., 2019). Classical and non-classical RNA-binding domains (RBDs) were detected from the drought stress RB-proteome identified in this study using pfam (http://pfam.xfam.org). RBPs and candidate RBPs were classified, as described previously (Beckmann et al., 2015). Co-expression for functional and data correlation analysis of selected up- and down- regulated proteins was performed using ATTED database (http://atted.jp).

#### Biophysical Characteristics and Sequence Topographies Analyses

Analyses of biophysical properties including length of proteins (number of amino acids), isoelectric points (p*I*) and hydrophobicity were performed using R (version 3.3.1). Amino acid composition enrichment between the drought stress responsive RBPome and input total proteome as reference as the background set was determined using the web-based composition profiler program (http://www.cprofiler.org/) using default setting and ordering amino acids by hydrophobicity (Kyte-Doolittle) (Vacic et al., 2007). Significance level was assessed using Bonferroni correction. Length and sequences of amino acids were retrieved from TAIR (https://www.arabidopsis.org/tools/bulk/sequences/index.jsp), the p*I* were obtained from TAIR (https://www.arabidopsis.org/tools/bulk/protein/index.jsp) and hydrophobicity values were calculated using the GRAVY calculator (http://www.gravy-calculator.de). The biophysical characteristics and sequence topographies distribution biases were assessed using R packages, as outlined previously (Reichel et al., 2016). Amino acid motif enrichment from the spliceosome and SG RBPs relative to the published RBP repertoire (Köster et al., 2017) and input proteome reference as background were analyzed using the Discriminative Regular Expression Motif Elicitation (DREME, http://meme-suite.org/tools/dreme) (Bailey, 2011) an interface in MEME suite version 4.12.0 (http://meme-suite.org) using an *E*-value threshold of 0.05.

#### Evolutionary Conservation of Drought Responsive Spliceosome and SG Proteins

To understand the conservation and potentially, the role of drought responsive RBPs associated with spliceosome and SGs, InParanoid version 8 (http://inparanoid.sbc.su.se/cgi-bin/index.cgi, Sonnhammer and Ostlund, 2015) was used to identify their predicted orthologs among selected dicots (*Glycine max, Solanum lycopersicum, Vitis vinifera*), monocots (*Brachypodium distachyon, Hordeum vulgare, Oryza sativa, Sorghum bicolor*), *Saccharomyces cerevisae, Drosophila melanogaster, Caenorhabditis elegans, Mus musculus*, and *Homo sapiens*. Here, a two-way prediction was possible. The InParanoid program generates ortholog groups that include all inparalogs with scoring below 0.05, which is achieved by using clustering rules based on genome-wide pairwise sequence similarity matches between two species (Sonnhammer and Ostlund, 2015). Phylogenetic tree construction was performed using the phylogeny web service (Phylogeny.fr), which utilizes multiple sequence alignment of as sequences from each organism and *BLAST-*EXPLORER to build datasets for phylogenetic analysis (Dereeper et al., 2008, 2010). The ScanProsite (http://prosite.expasy.org/) was used for motif and copy number assignment.

## Results and Discussion

### Spliceosome Components and Transcriptional Regulation During Drought Stress

A distinct drought-responsive RNA-binding proteome has already been established (Marondedze et al., 2019). To gain further insights into the drought-responsive RNA-binding proteome, we interrogated changes in the spliceosome components, an important step in the genesis of RNA processing and modifications, to better understand the regulation of post-transcriptional stress responses at the systems level.

Among the 1408 proteins detected in at least two biological replicates, 74 are associated with the spliceosome pathway. Of the 74 proteins, 23 are significantly (*p-*value ≤ 0.05) responsive to drought stress as compared to the controls and 21 proteins were detected to associate with mRNA either at 1 or 4 h post-treatment (Table 1, Supplementary File 1).

**Table 1:** Spliceosome and stress granule associated drought stress responsive RBPs.

		**1 h**		**4 h**	
**Accession**	**Description**	**log_**2**_FC**	**adj *p*-value**	**log_**2**_FC**	**adj *p*-value**
**Spliceosome associated proteins**					
AT1G74230	Glycine-rich RNA-binding protein 5	−2.56	0.004		
AT5G02530	RNA-binding (RRM/RBD/RNP motifs) protein	−2.11	0.037		
AT2G23930	Probable small nuclear ribonucleoprotein G	−2.09	0.043		
AT4G00830	RNA-binding (RRM/RBD/RNP motifs) protein	−1.82	0.006		
AT1G03457	RNA-binding (RRM/RBD/RNP motifs) protein	−1.77	0.034		
AT4G16830	Hyaluronan/mRNA binding (ATRGGA)	−1.75	0.038	−2.41	0.02
AT1G49760	Poly(A) binding protein 8	−1.39	0.039		
AT2G23350	Poly(A) binding protein 4	−1.37	0.043		
AT3G49430	SER/ARG-rich protein 34A	−1.21	0.030		
AT3G15010	RNA-binding (RRM/RBD/RNP motifs) protein	−1.20	0.009		
AT5G61030	Glycine-rich RNA-binding protein 3	−1.03	0.042		
AT5G04280	RNA-binding glycine-rich protein B3	−0.88	0.022		
AT2G37340	Arg/Ser-rich Zn knuckle-containing protein 33	−0.65	0.044		
AT2G13540	ABA HYPERSENSITIVE 1	0.74	0.006	1.80	0.04
AT2G33340	MOS4-associated complex 3B	0.81	0.040		
AT3G49601	mRNA splicing factor containing protein	0.93	0.041		
AT3G04610	RNA-binding KH domain-containing protein			−1.85	0.04
AT3G58570	P-loop nucleoside triphosphate hydrolase			−1.39	0.03
AT1G14170	RNA-binding KH domain-containing protein			−1.28	0.05
AT4G17520	Hyaluronan/mRNA binding			−1.17	0.00
AT1G60650	RNA-Binding glycine-rich protein B1			0.64	0.03
AT5G04430	Binding to TOMV RNA 1L			0.84	0.01
AT3G55460	SC35-like splicing factor 30			1.11	0.02
AT1G04510	MOS4-associated complex 3A	X		X	
AT1G55310	SC35-like splicing factor 33	X		X	
AT1G80930	MIF4G domain-containing protein	X		X	
AT2G27100	C2H2 zinc-finger protein SERRATE (SE)	X		X	
AT2G43810	Small nuclear ribonucleoprotein family protein	X		X	
AT3G13570	SC35-like splicing factor 30A	X		X	
AT3G26560	ATP-dependent RNA helicase, putative	X		X	
AT5G48870	Small nuclear ribonucleoprotein family protein	X		X	
AT1G09770	Cell division cycle 5	X			
AT4G02840	Small nuclear ribonucleoprotein family protein	X			
AT4G30330	Small nuclear ribonucleoprotein family protein	X			
AT4G38780	Pre-mRNA-processing-splicing factor	X			
AT5G16780	SART-1 family	X			
AT5G61140	U5 small nuclear ribonucleoprotein helicase	X			
AT1G09140	Serine/Arginine protein 30			X	
AT1G71310	cobalt ion binding			X	
AT1G76860	Small nuclear ribonucleoprotein			X	
AT4G14342	Splicing factor 3B subunit 5			X	
AT5G44200	CAP-binding protein 20			X	
AT1G03330	Small nuclear ribonucleoprotein	X^c^		X^c^	
AT4G35785	RNA-binding (RRM/RBD/RNP motifs)	X^c^		X^c^	
**Stress granule associated proteins**					
AT1G11650	RNA-binding 45B	−2.60	0.029		
AT3G07810	RNA-binding (RRM/RBD/RNP motifs) protein	−2.32	0.032		
AT5G54900	RNA-binding protein 45A	−2.21	0.030		
AT1G49600	RNA-binding protein 47A	−2.19	0.038		
AT4G27000	RNA-binding 45C	−2.05	0.005		
AT5G43960	Ras-GAP SH3 domain-binding protein	−2.00	0.022	−0.98	0.03
AT3G19130	RNA-binding protein 47B	−1.96	0.045		
AT4G27500	Proton pump interactor 1	−1.89	0.045		
AT1G49760	Poly(A) binding protein 8	−1.39	0.039		
AT2G23350	Poly(A) binding protein 4	−1.37	0.043		
AT1G13020	Eukaryotic (Euk.) initiation factor 4B2	−1.19	0.021		
AT1G24510	TCP-1/cpn60 chaperonin	1.63	0.003		
AT5G20890	TCP-1/cpn60 chaperonin	1.79	0.007		
AT4G31880	Tudor/PWWP/MBT super protein	2.32	0.012		
AT1G04170	Euk. translation initiation factor 2 gamma			3.58	0.00
AT2G40290	Euk. translation initiation factor2 subunit 1			0.75	0.02
AT1G02920	Glutathione S-transferase 7	X		X	
AT1G02930	Glutathione S-transferase 6	X		X	
AT1G27310	Nuclear transport factor 2A	X		X	
AT1G30230	Eukaryotic initiation factor 1B BETA 1	X		X	
AT1G45145	Thioredoxin H-type 5	X		X	
AT1G62380	1-aminocyclopropane-1-carboxylic oxidase2	X		X	
AT1G78570	Rhamnose biosynthesis 1	X		X	
AT4G34050	Caffeoyl coenzyme A	X		X	
AT3G03920	H/ACA RNP complex, Gar1/Naf1 protein	X			
AT3G17390	S-adenosylmethionine synthetase	X			
AT1G09640	Translation elongation factor EF1B			X	
AT1G09780	2,3-biphosphoglycerate mutase 1			X	
AT1G71770	Poly(A) binding protein 5			X	
AT2G42520	Glutathione S-transferase PHI 2			X	
AT3G09440	Heat shock protein 70			X	
AT4G02520	RNA helicase 37			X	

*X- proteins detected only after stress treatment in the mRNA interactome*.

*X^c^- proteins detected only in the controls and absent in the treated samples*.

**Figure 1:**
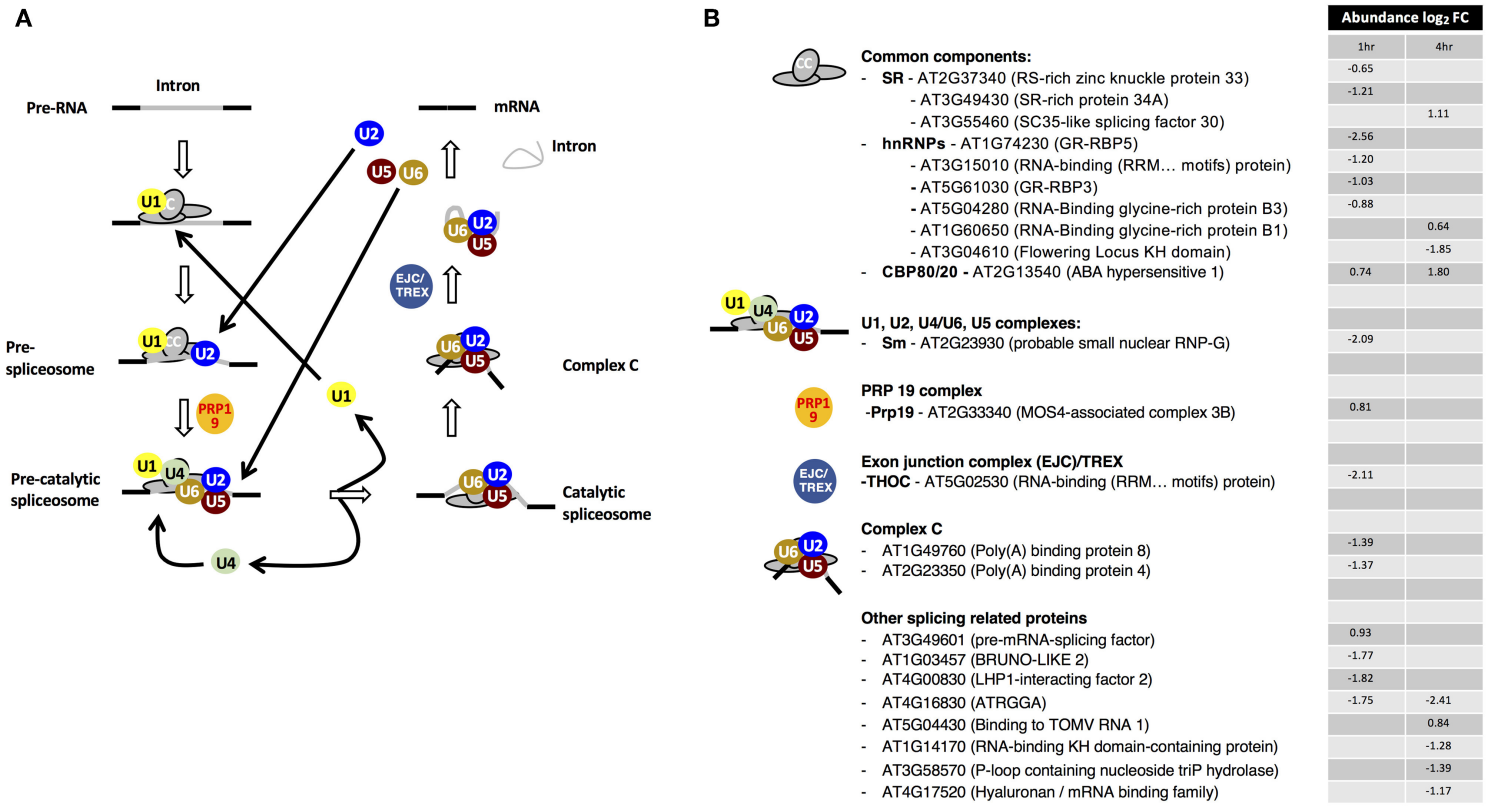
Drought stress-dependent responses of the spliceosome components. **(A)** Dynamic reorganizations and components of the spliceosome assembly system. **(B)** Drought stress-dependent changes in the RNA interaction status of spliceosome components. Adapted from Marondedze et al. (2016a).

Of the 23 enriched spliceosome components, ten proteins belong to the common components (CC) category (Figure 1). The second dominant group comprises of other splicing-related proteins, with eight proteins. Overall 17 proteins are decreasing in abundance at 1 h and/or 4 h post-treatment. In the serine/arginine-rich (SR) category, serine/arginine-rich splicing 30 or SC35-like splicing factor 30 (SCL30) is increasing in abundance at 4 h while RS-rich zinc knuckle-containing protein 33 (AtRSZ33, AT2G37340) and SR-rich protein 34A (SRp34A, AT3G49430) are decreasing in abundance at 1 h after drought stress treatment (Figure 1B). The SR proteins play a role in the constitutive splicing and selection of alternative splice sites in plants and animals, a central mechanism to generate proteome diversity and regulating gene expression (Zhou et al., 2002). In plants, alternative splicing profiles are modulated by environmental stress, hormones and various organ developmental stages (Iida et al., 2004; Palusa et al., 2007). For example, under high light irradiation and salinity, transcript levels of SCL30 and AtSR45A increase and their splicing patterns are altered (Tanabe et al., 2007). In the current study, SCL30 is increasing in abundance 4 h post-treatment in the mRNA interactome suggesting that the role of SCL30 is enhanced under drought stress and conceivably indicating posttranscriptional gene regulatory activity. Overall, SR proteins have been reported to contribute to alternative splicing by affecting splice sites selection in a concentration- and phosphorylation-dependent manner and seem to control this process in a developmental-regulated, tissue-specific and stress-responsive manner (Duque, 2011).

Two of the six heterogeneous nuclear ribonucleoproteins (hnRNPs), RNA-binding glycine-rich protein B1 (AtRZ-B1, AT1G60650) and B3 (AtRZ-1C, AT5G04280) have been reported to function as pre-mRNA splicing regulators in response to cold stress and water deprivation (Kim et al., 2010). At transcriptional level, AtRZ-1B decreased over time while AtRZ-1C increased in response to drought stress (Kim et al., 2010). In contrast, at RBP level after stress exposure, the abundance of AtRZ-1B increases at 4 h while that of AtRZ-1C decreases at 1 h (Table 1, Figure 1B).

Notably, established ABA-responsive proteins were detected as part of the spliceosome pathway and these include ABA hypersensitive 1 (also known as cap binding protein 80 (CBP80); AT2G13540) that increased in mRNA association at both 1 and 4 h post-treatment, hyaluronan (AtRGGA, AT4G16830) and RNA-binding K homology (KH) domain-containing protein (AT1G14170) whose abundances decreased over time (Figure 1B). In addition, cap binding protein 20 (CBP20) was detected only after stress treatment. CBP80/20 complex of the CC class is a heterodimeric complex that is essential for RNA metabolism through binding to the mRNA cap structure (Hugouvieux et al., 2001; Kierzkowski et al., 2009). Arabidopsis CBP20 has a role in ABA regulation and drought stress (Papp et al., 2004) while its partner CBP80 is known to modulate early ABA signal transduction (Hugouvieux et al., 2001) and acting in the flowering pathway (Kuhn et al., 2008). It is noteworthy that in the *abh1 (cpb80)* mutant, plants show ABA-hypersensitive stomatal closure, reduced wilting under drought stress and transcripts implicated in ABA signaling were down-regulated. Cytosolic calcium increased in the *abh1* guard cells suggesting amplification of early ABA signaling response (Hugouvieux et al., 2001). It has also been reported that CBP20 and CBP80 are stabilized by ABA via a post-translational mechanism and that these proteins participate in ABA induction of miR159 during seed germination resulting in increased abiotic stress tolerance (Kim et al., 2008). Taken together, the increase in CBP80/20 complex proteins, similarly to reported ABA responses, implicate them in the post-transcriptional regulation of drought stress responsive genes.

AtRGGA, an RBP localized in the perinuclear space and cytoplasm, has been shown to increase in expression in Arabidopsis seedlings in response to ABA and PEG treatment (Ambrosone et al., 2015). Transgenic plants overexpressing AtRGGA showed more tolerance to ABA, drought and salt stresses as indicated by lower rate of water lose in detached leaf assays when compared to control untransformed plants. The *rgga* mutant showed high sensitivity to ABA and osmotic stress, suggesting that AtRGGA participates in ABA-dependent mechanisms of salt and drought stress responses (Ambrosone et al., 2015). Expression profile analysis using genecat (https://www.arabidopsis.org) shows that AtRGGA is highly expressed in seeds, particularly under dehydration. In the current study, we observe that at RBP level, the abundance of AtRGGA decreases in response to drought stress although at the RNA level it has been observed to increase. These findings suggest a reduction in the modulation of AtRGGA RNA targets possibly to facilitate its regulatory function in enhancing tolerance to drought stress.

Furthermore, six of the 23 significantly changing proteins are increasing in abundance during drought stress. Co-expression analysis of the six proteins reveal that 36 proteins of the spliceosome are co-expressed with at least one of the six proteins. Among the 36 proteins, 10 proteins are co-expressed with at least two of the six up-regulated proteins. Three of the proteins, modifier of suppressor of non-expresser of pathogenesis-related genes 1, 4 (MOS4)-associated complex 3B, SCL30 and SCL33 are increasing in abundance after drought stress while the remaining seven are decreasing. Notably, 12 of the co-expressed proteins are known components of the stress granules, suggesting a potential crosstalk between spliceosome function and translational arrest, which are two distant biological processes in stress response. A global gene ontology analysis of the 300 co-expressed proteins for each of the six up-regulated proteins show an enrichment bias on biological processes such as “RNA metabolic process,” “primary metabolic process,” “methylation,” “chromosome organization,” and “flower development” depicting that various key processes pre- and post- transcription are associated with drought stress responsive spliceosome components. We therefore postulate that the spliceosome is a key regulatory component that contributes to stress-dependent transcriptional regulation.

### Stress Granule Components and Their Role in Drought Stress

In addition, we set out to detect stress granule components and changes in their abundance. We observed that a third (12 proteins) of the proteins co-expressed with spliceosome proteins, which are increasing in abundance during drought stress are classical components of SGs. Seven of the 12 proteins are also either increasing in abundance or detected to interact with mRNA after drought stress treatment (Supplementary File 2). Interestingly, of the seven, three proteins namely, TCP-1/chaperonin 60 (AT1G24510 and AT5G20890), and Tudor (AT4G31880), are co-expressed with CBP80, while eukaryotic translation initiation factor 2δ (AT1G04170), eukaryotic translation initiation factor 2 subunit 1 (AT2G40290) and TCP-1/chaperonin 60 (AT5G20890) are co-expressed with the protein Binding to TOMV RNA 1L (AT5G04430). Binding to TOMV RNA 1L is also co-expressed with additional four SG proteins namely, PAB4 (AT2G23350), PAB8 (AT1G49760), RBP 47A (AT1G49600) and Ras-GAP SH3 domain-binding protein [also called nuclear factor 2 (AT5G43960)] whose abundances decrease upon drought stress. However, PAB4 and PAB8 are shared between spliceosome and SG potentially suggesting a common post-transcriptional regulation function. The remaining proteins glutathione S-transferase PHI 2 (AT2G42520) and nuclear transport factor 2A (AT1G27310) are co-expressed with RNA-binding glycine-rich protein B1 (AT1G60650) and the splice factor SCL30, respectively. This relationship signifies an important potential crosstalk that may exist between the spliceosome and SG assembly during stress responses.

In order to gain a global picture on SG composition, we looked at the entire drought stress responsive mRNA binding proteome data to see how many SG proteins cropped up in the data and how they are modified under drought stress. This led to the identification of 32 SG associated proteins including the 12 described above (Table 1, Supplementary File 1). Seventeen of the SG components significantly changed in abundance upon stress compared to the control set. SGs are an essential part of the response to environmental stresses through reversible translational suppression that acts as regulators of mRNA storage and stability e.g., during oxidative stress (Keene, 2007), heat stress (Weber et al., 2008), and hypoxia (Sorenson and Bailey-Serres, 2014). SG components regulate translational initiation phase of recovery after environmental stresses.

Co-expression analysis was performed on a set of SG proteins including rhamnose biosynthesis 1 (AT1G78570), RBP45B (At1g11650), and Ras-GAP SH3 domain-binding protein (Supplementary File 3). Notably, five proteins among the rhamnose biosynthesis 1 co-expressed genes are part of the stress granule components. Just like rhamnose, four proteins namely, 2,3-biphosphoglycerate mutase 1, Heat shock protein 70, S-adenosylmethionine synthetase and caffeoyl coenzyme A show drought stress induced mRNA interactions. Rhamnose biosynthesis 1 is a protein involved in the biosynthesis of rhamnose, a major monosaccharide component of pectin. Together with 2,3-biphosphoglycerate mutase 1, Heat shock protein 70 and caffeoyl coenzyme A, they have been recently identified as part of the SG but have no known RBDs (Kosmacz et al., 2019). In turn, RBP45B, a SG protein, is co-expressed with proteins that are part of the spliceosome. Similarly to RBP45B nearly 90% of its co-expressed counterparts (from Table 1) are also decreasing in abundance upon drought stress treatment (Supplementary File 3). Ras-GAP SH3 domain-binding protein, another notable component of the SG is co-expressed 17 SG and spliceosome components and 11 of these proteins are decreasing in abundance just like itself (Supplementary File 3). Overall, co-expression analysis demonstrates the strong connectivity of networks and events associated with drought stress and in particular among proteins that are involved in spliceosome and stress granule formation and/or maintenance. In addition, identification of the spliceosome, and SG associated RBPs adds confidence to our experimental system and it sheds new light on the interrelation of biological processes in mRNA association during drought stress at the systems level.

### Dynamic Abundance of Spliceosome and Stress Granule RBPs

**Figure 2:**
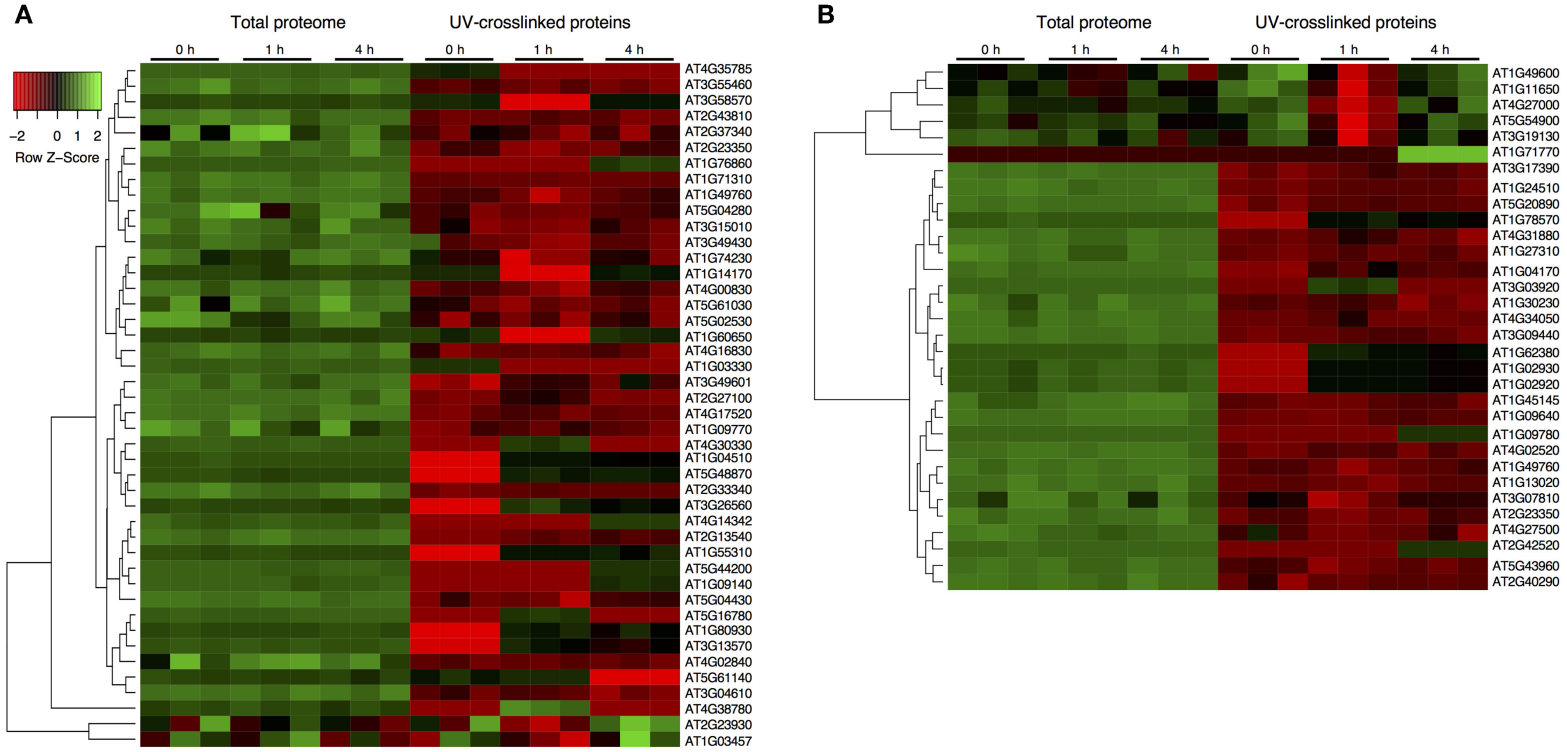
Dynamic characterization of the spliceosome and stress granule RBPs after drought stress. **(A)** Protein abundance from total soluble proteome and UV-crosslinked samples for the spliceosome components. **(B)** Protein abundance for stress granule associated proteins from total soluble proteome and UV-crosslinked samples. Abundance z-score was normalized within each sample extraction type. Hierarchical clustering across all samples is shown on the left, and on the right are the protein accession numbers. Individual RBPs with a significant increase upon UV crosslinked, stress exposure and compared to their respective UV crosslinked samples are highlighted in blue.

Quantifying protein abundance in UV crosslinked samples and total soluble proteins in the cell lysates from the same sample, allowed us to determine changes in RNA-binding that were discrete from coexisting changes in the total protein abundance. In general, RBPs show distinct abundances compared to the total proteins (Figure 2). In the spliceosome, most RBPs decreased in abundance relative to the total protein suggesting that although the RNA association is modified, the overall abundance of the proteins is not (Figure 2A). Important to note that RBPs such as CBP80, SC30 and Binding to TOMV RNA 1L, that show a significant increase in RBP level after stress are decreasing in abundance relative to their total protein level, indicating an increase in their RNA association rather than at the translational level. Notably, stress granule components, PAB5 (AT1G71770), RBP47A (AT1G49600), RBP45B, and RBP45A (AT5G54900) increase in abundance at RBP relative to the total protein levels. Essentially, 11 RBPs increase in their abundance for RNA interaction compared to the control upon RBP enrichment potentially increasing the mRNA occupancy (Figure 2B). Remarkably, the changes in UV-enriched protein abundance often do not correlate with variations in total protein abundance, signifying that specific RBPs bind RNA differentially during stress.

### Domain Organization of Spliceosome and SG Associated Drought Stress Responsive RBPs

Analysis of domain diversity was performed using Prosite (http://prosite.expasy.org). Majority of the proteins contain at least one of the classical RBDs (Supplementary File 4) including the RNA recognition motif (RRM) domain harbored by 27 proteins. The RRM domain is the most dominant classical RBD and its dominance among drought stress responsive proteins may suggest a preferential or specific stress response control mechanism. Conceivably, this expands the protein function to the transcriptional regulation of responses to abiotic stress. At 1 h post-treatment only, abundances of all the RRM domain-containing proteins including glycine-rich RBP5, RNA binding family proteins (AT5G02530, AT4G00830, AT1G03457), PAB4, PAB8, RBP45A, RBP45B, RBP45C, and RBP47B decreased (Table 1). It remains unresolved whether domain architecture and/or domain organization of an RBP affect RNA targets in response to a drought stress but we hypothesize that environmental stress responses may operate via RBP with a particular domain architecture and in turn influence their RNA targets.

### Analyses of Amino Acid Motifs, Biophysical Features and Sequence Topology

**Figure 3:**
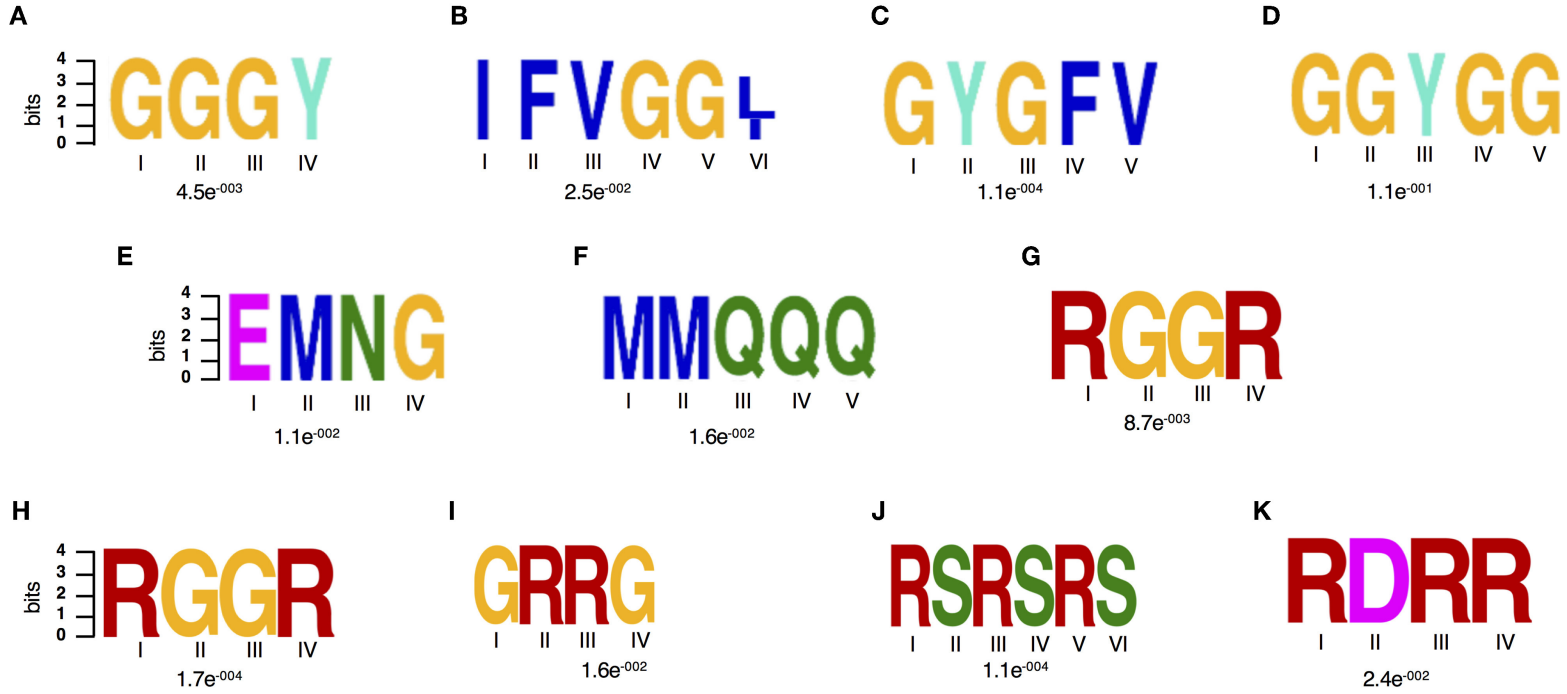
Amino acid motif enrichment. **(A–F)** Sequence features of the drought stress responsive spliceosome and stress granule associated RBPs. **(A,B)** Two significantly enriched amino acid motifs against the RBP repertoire (Köster et al., 2017), **(A–G)** Seven enriched amino acid motifs against the input proteome reference and **(H–K)** enriched motifs from the spliceosome specific data set as analyzed by the DREME software, which is part of the MEME suite.

In order to gain further insights in what could be driving this targeted change on RBPs particularly their differential accumulation and RNA interaction, we characterized their amino acid sequence complexity features. Enriched amino acid sequence motifs were computed against the published RBP repertoire (Köster et al., 2017) and then the input proteome reference using the DREME software, which is part of the MEME suite (Bailey, 2011). Two amino acid motifs were significantly (*E*-value threshold ≤0.05.) enriched against the background of the RBP repertoire (Figures 3A,B) and six motifs were detected as significantly enriched against the input proteome (Figures 3A–G). Enrichment is biased toward glycine-rich (GR) motifs that have been reported to play a role in several RNA-associated processes (Figures 3A–D) (Thandapani et al., 2013). The GR motifs detected include the GGGY, GYGFV, IFVGG[LI], and GGYGG. The GGGY motif belongs to the GR repeat class I category and is detected in GR containing proteins that act as signal peptides and play a role in pre-mRNA processing, for example the ATP-dependent RNA helicases. The motifs, GYGFV and IFVGG[LI], are classical conserved consensus sequences of the RRM domains of RNP1 and 2, respectively (Lorkovic and Barta, 2002). Motifs GGGY and GYGFV have been detected in dehydrins, a group of evolutionarily conserved GR hydrophilic “late embryogenesis proteins,” which are responsive to ABA treatment, dehydration, salinity or cold stress (Allagulova Ch et al., 2003). Other motifs enriched among the spliceosome and SG drought stress responsive are EMNG and MMQQQ (Figures 3F–G). Interestingly, when we consider only spliceosome proteins, we identified distinct motifs including two GR (RGGR and GRRG), an RS (RSRSRS) and RDRR motifs (Figures 3H–K) of which RGGR is consistently detected.

**Figure 4:**
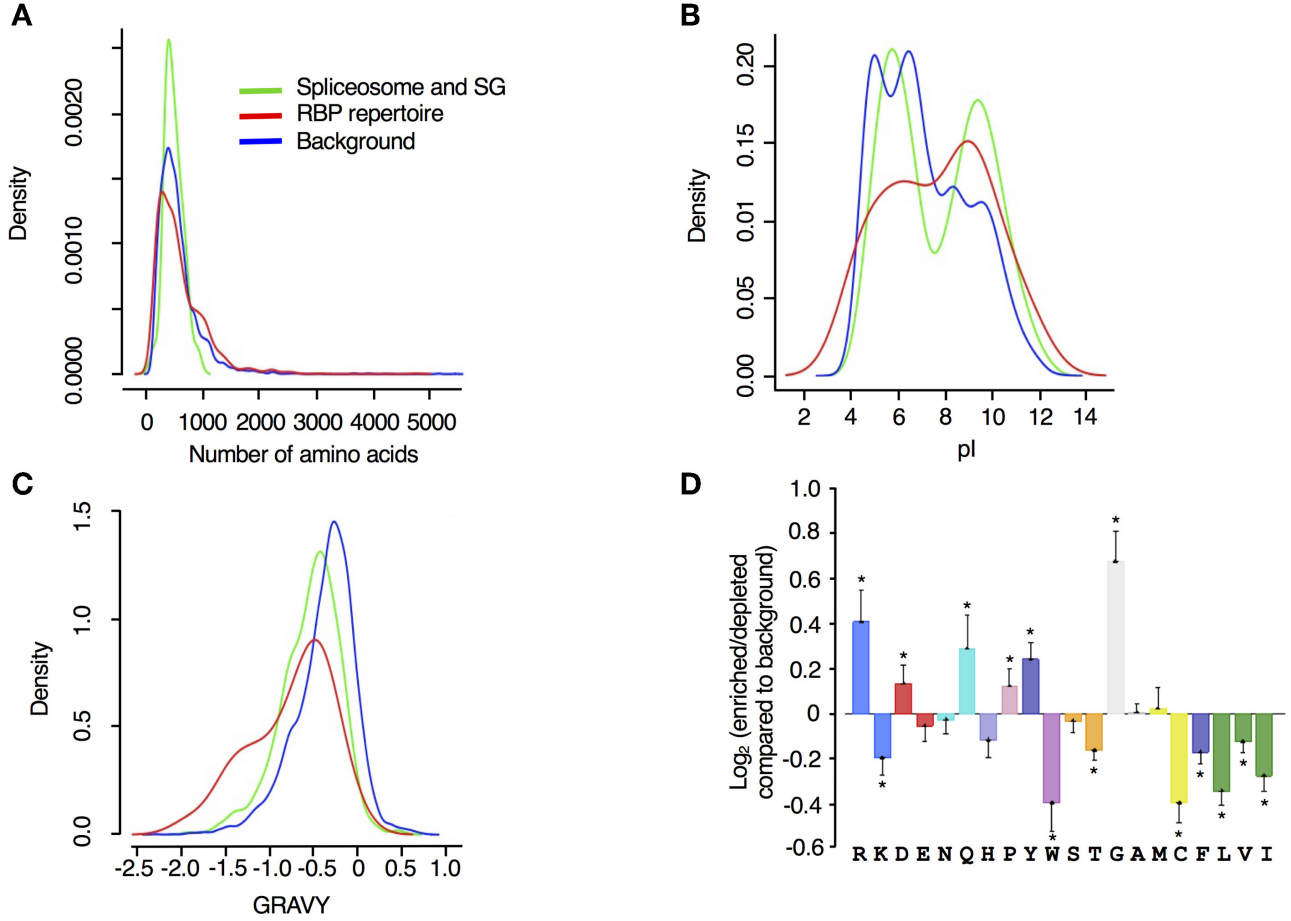
Biophysical features of drought stress responsive spliceosome and stress granule associated RBPs. Density of **(A)** protein length (number of amino acids), **(B)** isoelectric point (p*I*) and **(C)** hydrophobicity (gravy) were analyzed for spliceosome and stress granule associated proteins responsive to drought stress (green), RBP repertoire (Köster et al., 2017) (red) and input proteome from controls that are used as input or background (blue). **(D)** Log_2_ enrichment of amino acid residues in the spliceosome and stress granule associated proteins responsive to drought stress, determined using the composition profiler (http://www.cprofiler.org/). The significance of enrichment or depletion was tested by a two-sample *T*-test and amino acids that are significantly enriched or depleted (*p* ≤ 0.001) compared to the control background (*N* = 5630) are marked with an asterisk.

Examining the biophysical and amino acid characteristics of the spliceosome and SG associated drought stress response proteins, we observed that the two reference datasets span the whole spectrum of protein sizes (represented as number of amino acids in Figure 4A), while the spliceosome and SG associated proteins were generally <1200 amino acid residues long. The majority of proteins in all datasets contain <1000 aa and some inclination toward longer proteins was detected among the RBP repertoire (Figure 4A). However, this analysis was not significant probably due to the low number of proteins in our dataset compared to the background samples. Compared to the input reference, RBP repertoire exhibited an isoelectric point distribution trend that is skewed toward higher alkaline isoelectric points while spliceosome and SG proteins displayed two distinct peaks one in the acidic and one in the alkaline isoelectric points (Figure 4B). The RBP repertoire, spliceosome and SG-associated proteins exhibited bias toward lower hydrophobicity than the input proteome (Figure 4C). For these parameters, a similar trend was observed with regards to the drought stress responsive RBPs, in particular for classical RBPs and proteins whose association with RNA has been established, as previously described (Marondedze et al., 2019).

Amino acid distribution and enrichment between the reference input, spliceosome and SG associated proteins was determined using the composition profiler program (http://www.cprofiler.org/). Positive amino acids with polar side chains that have high affinity for RNA were enriched among the spliceosome and SG associated proteins and these include arginine (R), glutamine (Q), and aspartate (D) (Figure 4D). Glycine (G), an aa that is known to interact strongly with guanine (Lejeune et al., 2005), exhibited the highest enrichment. In contrast, aromatic, and hydrophobic aa, isoleucine (I), leucine (L), and valine (V), were underrepresented (Figure 4D). Also underrepresented, are amino acids with aliphatic side chains [phenylalanine (F) and tryptophan (W)]. Proline (P) was significantly enriched and has been reported as strongly enhanced in highly disordered protein regions, a central concept for RBPs (Sysoev et al., 2016). We also noted that a similar set of amino acids were enriched in the total RBPs responsive to drought stress with the exception of R and P, that are significantly enriched in the drought stress responsive spliceosome and stress granule proteins (Marondedze et al., 2019). Overall, these molecular and biophysical findings reflect previously reported characteristics of mRNA interactomes and confirm the properties of mRNA recognition. However, the drought stress response stimulus seems to favor a specific set of the spliceosome complex proteins that have specific characteristics as indicated by these biophysical characteristics, e.g., strong enrichment of aa like glycine, highly alkaline isoelectric points and bias toward relatively short length proteins.

### Conservation of Spliceosome and SG Associated drRBPs Across Different Species

Using Paranoid8 and spliceosome and SG proteins that are significantly regulated during drought stress, 24 proteins are predicted to have orthologs in dicotyledons and 26 in monocotyledons. Most of the orthologs are detected in barley (*Hordeum vulgare*) and rice (*Oryza sativa*) (Table 2, Supplementary File 5). Only 13 Arabidopsis proteins had orthologs detected in all the plant species examined in this study and three proteins had no orthologs in any species examined. The general trend of high sequence identity on predicted orthologs across the plant kingdom could serve as positive criteria in engineering increased drought tolerance in crop plants. In addition, 25 proteins were predicted to have orthologs in either *Homo sapiens, Mus musculus, Drosophila melanogaster, Caenorhabditis elegans*, or *Saccharomyces cerevisae*. Notably, the eight proteins with orthologs in yeast might indicate an ancient origin of RBP dependent processes.

**Table 2:** Orthologs of Arabidopsis enriched drought stress responsive RBPs acquired using InParanoid8 (see Supplementary File 4).

**Species**	**Number of proteins/orthologs**
Arabidopsis thaliana^*^	34
Glycine max^*^	24
Solanum lycopersicum^*^	21
Vitis vinifera^*^	22
*Brachypodium distachyon*^†^	22
*Hordeum vulgare*^†^	22
*Oryza sativa*^†^	22
*Sorghum bicolor*^†^	21
*Drosophila melanogaster*	14
*Caenorhabditis elegans*	13
*Homo sapiens*	18
*Mus musculus*	20
*Saccharomyces cerevisae*	8
Common in all plants only	13

* *Dicots*,

† *Monocots*.

**Figure 5:**
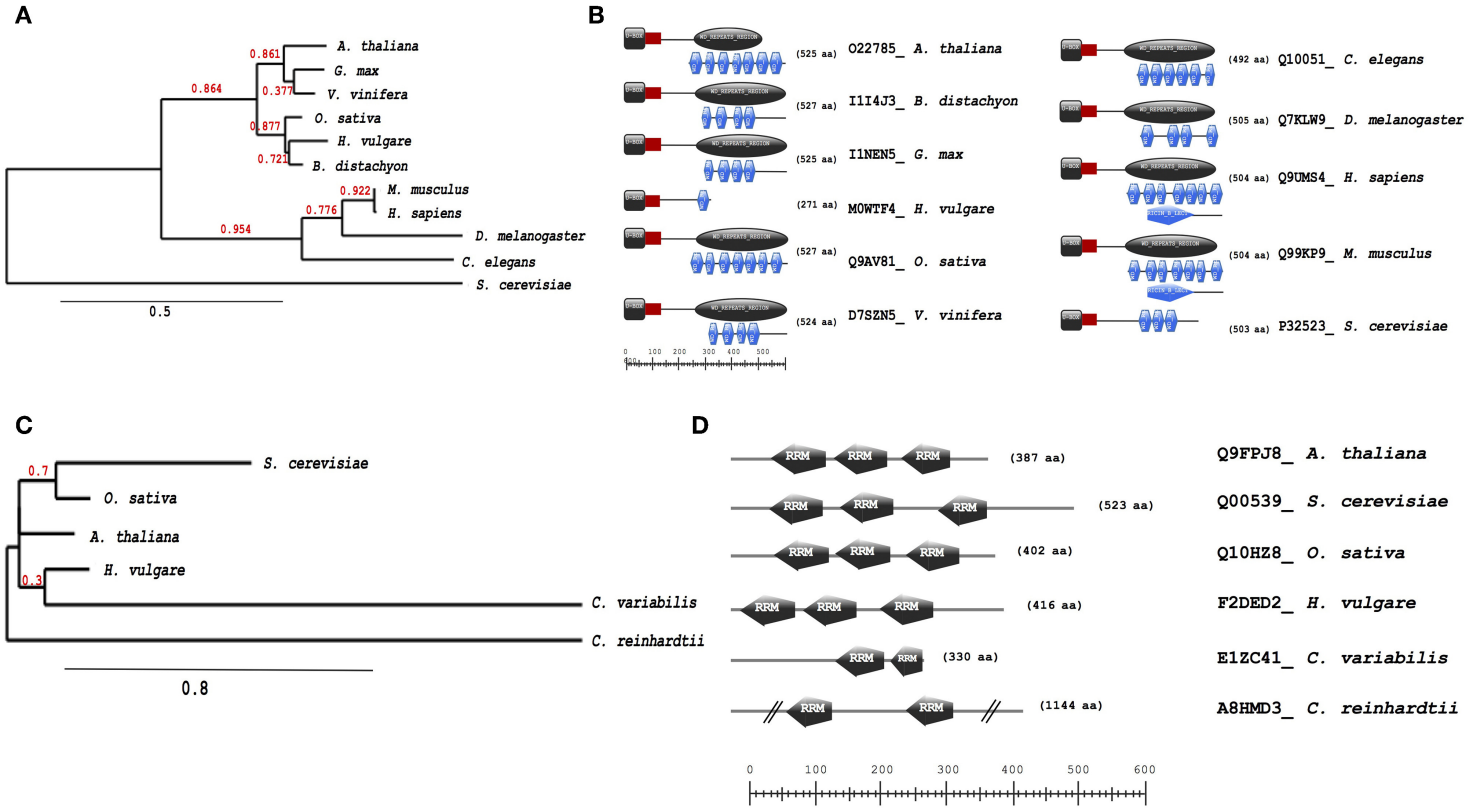
Evolutionary conservation of pre-mRNA processing factor 19 and polyadenylate-binding protein RBP45A. Phylogenetic and motif or domain copy numbers of pre-mRNA processing factor 19 (PRP19) **(A,B)** and polyadenylate-binding protein RBP45A **(C,D)**. Phylogenetic tree construction was performed using the phylogeny web service (Phylogeny.fr and the motifs and copy number assignments were performed using the ScanProsite (http://prosite.expasy.org/).

**Figure 6:**
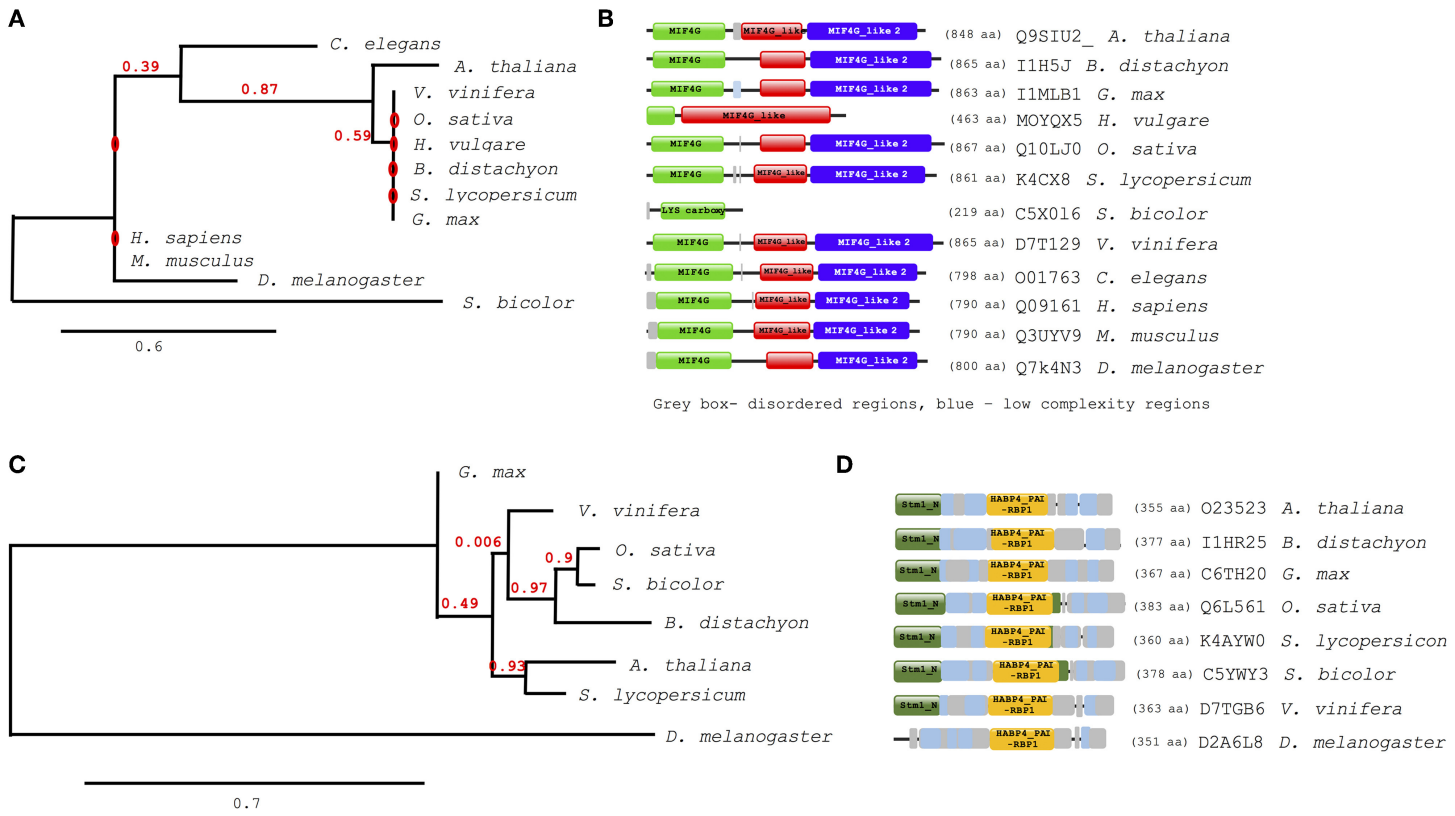
Evolutionary conservation of ABA hypersensitive 1 (CPB80) and AtRGGA (hyaluronan). Phylogenetic and motif or domain copy numbers of CBP80 **(A,B)** and AtRGGA **(C,D)**. Phylogenetic tree construction was performed using the phylogeny web service (Phylogeny.fr and the motifs and copy number assignments were performed using the ScanProsite (http://prosite.expasy.org/).

Further analysis of evolutionary trends of the Arabidopsis drought responsive spliceosome and SG associated protein orthologs across different selected species was performed using a combination of phylogenetics, protein sequence motif, and copy number. We observed that the pre-mRNA processing factor 19 (PRP19, AT2G33340), also termed MOS4-associated complex 3B, has orthologs in all of the species blasted (Supplementary File 5), ranging from yeast to higher plants and animals. The Arabidopsis protein is closely related to its orthologs from *Vitis vinifera* and *Gylcine max* (Figure 5A). This PRP19 protein is an ubiquitin-protein ligase, containing WD-40 repeats, that is involved in pre-mRNA splicing. Its role is conserved from yeast to higher eukaryotes including plants. It acts as a central component of the PRP19-associated complex that stabilizes the spliceosome structure after dissociation of the U4 snRNA, a role that is mediated by the WD-40 repeats (Smith et al., 1999; Ohi et al., 2003). In plants, PRP19 was postulated to regulate defense responses via transcriptional control (Monaghan et al., 2009). The PRP19 domains are present and highly conserved in all species, however, the WD-40 repeats vary in copy numbers from one in barley to seven in Arabidopsis, rice, human and mouse (Figure 5B). Another protein worth noting is the RBP45A, which has orthologs in rice, barley, yeast, and in primitive photosynthetic organisms, including the green algae (*Chlamydomonas reinhardtii*, Figure 5C), indicating an ancient origin. The RRM domains are conserved thus retaining the function of the protein, although in algae only two RRM domains are detected while in yeast and plants three RRM domains are present (Figure 5D). Other proteins such as the CBP80 and the hyaluronan (AtRGGA) that have previously been shown to be involved in drought stress have no orthologs in yeast (Supplementary File 5). An ortholog of the Arabidopsis CBP80 is detected in barley and sorghum (*Sorghum bicolor*) with a varying domain architecture (Figure 6A). In the latter, the protein lacks the MIF4G motif that is implicated in mRNA splicing (Figure 6B). The observed differences in domain architecture and composition could in part be that barley and sorghum are in general drought stress tolerant crops that may differ in their ABA regulation compared to Arabidopsis, for example.

The orthologs of hyaluronan were found in plants and drosophila but not in yeast (Figure 6C). Its absence in yeast is not surprising, as yeast does not synthesis hyaluronan. The plant orthologs have two main domains, the Stm1_N and the HABP4_PAI-RBP1 (Figure 6D). The Stm1_N domain is present at the N-terminal of some RGG repeats of nuclear RBPs and associates with ribosomes and nuclear telomere cap complexes (Van Dyke et al., 2004). The HABP4 domain has been observed to bind RNA but with lower affinity than that for hyaluronic acid (Huang et al., 2000), while the PAI-RBP1 binds mainly to the mRNA of plasminogen activator inhibitor 1 and regulates mRNA stability (Heaton et al., 2001).

## Conclusion

Drought induces significant changes of the spliceosome. In turn, these changes reflect modifications of transcriptional program and hence responses to stress. In addition, identification of stress granule components points to a translational arrest induced by drought stress. The overlap of co-expressed proteins between the two distinct molecular processes, spliceosome function, and stress granule formation, suggests the presence of a systems level response and possibly crosstalk. Furthermore, the presence of conserved stress responsive RBPs indicates an ancient origin of these proteins and possibly evolutionarily conserved posttranscriptional regulation that operates during stress responses and adaptation. This study sets the foundation for future detailed and mechanistic approaches to elucidate dynamic changes and functional roles of RBPs and their binding RNAs under various environmental cues.

## Data Availability Statement

The datasets generated for this study can be found in the PRIDE repository accession PXD016883.

## Author Contributions

CM, CG, KL, and LT designed the research. CM performed all the experiments and data analysis. KL and LT provided technical assistance. CM and CG drafted manuscript. All authors participated in revising the manuscript and approved the final version.

### Conflict of Interest

The authors declare that the research was conducted in the absence of any commercial or financial relationships that could be construed as a potential conflict of interest.

## References

[B1] Allagulova Ch R. Gimalov F. R. Shakirova F. M. Vakhitov V. A. (2003). The plant dehydrins: structure and putative functions. Biochemistry 68, 945–951. 10.1023/A:1026077825584 14606934

[B2] Ambrosone A. Batelli G. Nurcato R. Aurilia V. Punzo P. Bangarusamy D. K.. (2015). The Arabidopsis RNA-binding protein AtRGGA regulates tolerance to salt and drought stress. Plant Physiol. 168, 292–306. 10.1104/pp.114.255802 25783413 PMC4424017

[B3] Anderson P. Kedersha N. (2006). RNA granules. J. Cell Biol. 172, 803–808. 10.1083/jcb.200512082 16520386 PMC2063724

[B4] Bailey T. L. (2011). DREME: motif discovery in transcription factor ChIP-seq data. Bioinformatics 27, 1653–1659. 10.1093/bioinformatics/btr261 21543442 PMC3106199

[B5] Baltz A. G. Munschauer M. Schwanhausser B. Vasile A. Murakawa Y. Schueler M.. (2012). The mRNA-bound proteome and its global occupancy profile on protein-coding transcripts. Mol. Cell 46, 674–690. 10.1016/j.molcel.2012.05.021 22681889

[B6] Beckmann B. M. Horos R. Fischer B. Castello A. Eichelbaum K. Alleaume A. M.. (2015). The RNA-binding proteomes from yeast to man harbour conserved enigmRBPs. Nat. Commun. 6:10127. 10.1038/ncomms10127 26632259 PMC4686815

[B7] Benjamini Y. Hochberg Y. (1995). Controlling the false discovery rate: a practical and powerful approach to multiple testing. J. Royal Stat. Soc. 57, 289–300. 10.1111/j.2517-6161.1995.tb02031.x

[B8] Buchan J. R. Parker R. (2009). Eukaryotic stress granules: the ins and outs of translation. Mol. Cell 36, 932–941. 10.1016/j.molcel.2009.11.020 20064460 PMC2813218

[B9] Castello A. Horos R. Strein C. Fischer B. Eichelbaum K. Steinmetz L. M.. (2013). System-wide identification of RNA-binding proteins by interactome capture. Nat. Protoc. 8, 491–500. 10.1038/nprot.2013.020 23411631

[B10] Chantarachot T. Bailey-Serres J. (2018). Polysomes, stress granules, and processing bodies: a dynamic triumvirate controlling cytoplasmic mRNA fate and function. Plant Physiol. 176, 254–269. 10.1104/pp.17.01468 29158329 PMC5761823

[B11] Cole C. N. (2001). Choreographing mRNA biogenesis. Nat. Genet. 29, 6–7. 10.1038/ng0901-6 11528378

[B12] Dereeper A. Audic S. Claverie J. M. Blanc G. (2010). Blast-explorer helps you building datasets for phylogenetic analysis. BMC Evol. Biol. 10:8. 10.1186/1471-2148-10-8 20067610 PMC2821324

[B13] Dereeper A. Guignon V. Blanc G. Audic S. Buffet S. Chevenet F.. (2008). Phylogeny.fr: robust phylogenetic analysis for the non-specialist. Nucleic Acids Res. 36, W465–W469. 10.1093/nar/gkn180 18424797 PMC2447785

[B14] Duque P. (2011). A role for SR proteins in plant stress responses. Plant Signal. Behav. 6, 49–54. 10.4161/psb.6.1.14063 21258207 PMC3122005

[B15] Heaton J. H. Dlakic W. M. Dlakic M. Gelehrter T. D. (2001). Identification and cDNA cloning of a novel RNA-binding protein that interacts with the cyclic nucleotide-responsive sequence in the Type-1 plasminogen activator inhibitor mRNA. J. Biol. Chem. 276, 3341–3347. 10.1074/jbc.M006538200 11001948

[B16] Huang L. Grammatikakis N. Yoneda M. Banerjee S. D. Toole B. P. (2000). Molecular characterization of a novel intracellular hyaluronan-binding protein. J. Biol. Chem. 275, 29829–29839. 10.1074/jbc.M002737200 10887182

[B17] Hugouvieux V. Kwak J. M. Schroeder J. I. (2001). An mRNA cap binding protein, ABH1, modulates early abscisic acid signal transduction in Arabidopsis. Cell 106, 477–487. 10.1016/S0092-8674(01)00460-3 11525733

[B18] Iida K. Seki M. Sakurai T. Satou M. Akiyama K. Toyoda T.. (2004). Genome-wide analysis of alternative pre-mRNA splicing in *Arabidopsis thaliana* based on full-length cDNA sequences. Nucleic Acids Res. 32, 5096–5103. 10.1093/nar/gkh845 15452276 PMC521658

[B19] Ivanov P. A. Nadezhdina E. S. (2006). Stress granules: RNP-containing cytoplasmic bodies springing up under stress. The structure and mechanism of organization. Mol. Biol. 40, 937–944. 10.1134/S0026893306060021 17209421

[B20] Kanehisa M. Sato Y. Morishima K. (2016). BlastKOALA and GhostKOALA: KEGG tools for functional characterization of genome and metagenome sequences. J. Mol. Biol. 428, 726–731. 10.1016/j.jmb.2015.11.006 26585406

[B21] Kedersha N. Anderson P. (2002). Stress granules: sites of mRNA triage that regulate mRNA stability and translatability. Biochem. Soc. Trans. 30(Pt 6), 963–969. 10.1042/bst0300963 12440955

[B22] Kedersha N. Stoecklin G. Ayodele M. Yacono P. Lykke-Andersen J. Fritzler M. J.. (2005). Stress granules and processing bodies are dynamically linked sites of mRNP remodeling. J. Cell Biol. 169, 871–884. 10.1083/jcb.200502088 15967811 PMC2171635

[B23] Keene J. D. (2007). RNA regulons: coordination of post-transcriptional events. Nat. Rev. Genet. 8, 533–543. 10.1038/nrg2111 17572691

[B24] Kierzkowski D. Kmieciak M. Piontek P. Wojtaszek P. Szweykowska-Kulinska Z. Jarmolowski A. (2009). The Arabidopsis CBP20 targets the cap-binding complex to the nucleus, and is stabilized by CBP80. Plant J. 59, 814–825. 10.1111/j.1365-313X.2009.03915.x 19453442

[B25] Kim S. Yang J. Y. Xu J. Jang I. C. Prigge M. J. Chua N. H. (2008). Two cap-binding proteins CBP20 and CBP80 are involved in processing primary MicroRNAs. Plant Cell Physiol. 49, 1634–1644. 10.1093/pcp/pcn146 18829588 PMC2722234

[B26] Kim W. Y. Kim J. Y. Jung H. J. Oh S. H. Han Y. S. Kang H. (2010). Comparative analysis of Arabidopsis zinc finger-containing glycine-rich RNA-binding proteins during cold adaptation. Plant Physiol. Biochem. 48, 866–872. 10.1016/j.plaphy.2010.08.013 20850334

[B27] Kimball S. R. Horetsky R. L. Ron D. Jefferson L. S. Harding H. P. (2003). Mammalian stress granules represent sites of accumulation of stalled translation initiation complexes. Am. J. Physiol. Cell Physiol. 284, C273–C284. 10.1152/ajpcell.00314.2002 12388085

[B28] Kosmacz M. Gorka M. Schmidt S. Luzarowski M. Moreno J. C. Szlachetko J.. (2019). Protein and metabolite composition of Arabidopsis stress granules. New Phytol. 222, 1420–1433. 10.1111/nph.15690 30664249

[B29] Köster T. Marondedze C. Meyer K. Staiger D. (2017). RNA-binding proteins revisited – the emerging Arabidopsis mRNA interactome. Trends Plant Sci. 22, 512–526. 10.1016/j.tplants.2017.03.009 28412036

[B30] Kuhn J. M. Hugouvieux V. Schroeder J. I. (2008). mRNA cap binding proteins: effects on abscisic acid signal transduction, mRNA processing, and microarray analyses. Curr. Top. Microbiol. Immunol. 326, 139–150. 10.1007/978-3-540-76776-3_8 18630751

[B31] Kwon S. C. Yi H. Eichelbaum K. Fohr S. Fischer B. You K. T.. (2013). The RNA-binding protein repertoire of embryonic stem cells. Nat. Struct. Mol. Biol. 20, 1122–1130. 10.1038/nsmb.2638 23912277

[B32] Lejeune D. Delsaux N. Charloteaux B. Thomas A. Brasseur R. (2005). Protein-nucleic acid recognition: statistical analysis of atomic interactions and influence of DNA structure. Proteins 61, 258–271. 10.1002/prot.20607 16121397

[B33] Liepelt A. Naarmann-de Vries I. S. Simons N. Eichelbaum K. Fohr S. Archer S. K.. (2016). Identification of RNA-binding proteins in macrophages by interactome capture. Mol. Cell. Proteomics 15, 2699–2714. 10.1074/mcp.M115.056564 27281784 PMC4974345

[B34] Lorkovic Z. J. Barta A. (2002). Genome analysis: RNA recognition motif (RRM) and K homology (KH) domain RNA-binding proteins from the flowering plant *Arabidopsis thaliana*. Nucleic Acids Res. 30, 623–635. 10.1093/nar/30.3.623 11809873 PMC100298

[B35] Marondedze C. Groen A. J. Thomas L. Lilley K. S. Gehring C. (2016a). A quantitative phosphoproteome analysis of cGMP-dependent cellular responses in *Arabidopsis thaliana*. Mol. Plant 9, 621–623. 10.1016/j.molp.2015.11.007 26658240

[B36] Marondedze C. Thomas L. Gehring C. Lilley K. (2019). Changes in the Arabidopsis RNA-binding proteome reveal novel stress response mechanisms. BMC Plant Biol. 19:139. 10.1186/s12870-019-1750-x 30975080 PMC6460520

[B37] Marondedze C. Thomas L. Serrano N. L. Lilley K. S. Gehring C. (2016b). The RNA-binding protein repertoire of *Arabidopsis thaliana*. Sci. Rep. 6:29766. 10.1038/srep29766 27405932 PMC4942612

[B38] Marondedze C. Turek I. Parrott B. Thomas L. Jankovic B. Lilley K. S.. (2013). Structural and functional characteristics of cGMP-dependent methionine oxidation in *Arabidopsis thaliana* proteins. Cell Commun. Signal. 11:1. 10.1186/1478-811X-11-1 23289948 PMC3544604

[B39] Marondedze C. Wong A. Groen A. Serrano N. Jankovic B. Lilley K.. (2014). Exploring the Arabidopsis proteome: influence of protein solubilization buffers on proteome coverage. Int. J. Mol. Sci. 16, 857–870. 10.3390/ijms16010857 25561235 PMC4307279

[B40] Merret R. Descombin J. Juan Y. T. Favory J. J. Carpentier M. C. Chaparro C.. (2013). XRN4 and LARP1 are required for a heat-triggered mRNA decay pathway involved in plant acclimation and survival during thermal stress. Cell Rep. 5, 1279–1293. 10.1016/j.celrep.2013.11.019 24332370

[B41] Monaghan J. Xu F. Gao M. Zhao Q. Palma K. Long C.. (2009). Two Prp19-like U-box proteins in the MOS4-associated complex play redundant roles in plant innate immunity. PLoS Pathog. 5:e1000526. 10.1371/journal.ppat.1000526 19629177 PMC2709443

[B42] Ohi M. D. Vander Kooi C. W. Rosenberg J. A. Chazin W. J. Gould K. L. (2003). Structural insights into the U-box, a domain associated with multi-ubiquitination. Nat. Struct. Biol. 10, 250–255. 10.1038/nsb906 12627222 PMC5881891

[B43] Ordonez N. M. Marondedze C. Thomas L. Pasqualini S. Shabala L. Shabala S.. (2014). Cyclic mononucleotides modulate potassium and calcium flux responses to H2O2 in Arabidopsis roots. FEBS Lett. 588, 1008–1015. 10.1016/j.febslet.2014.01.062 24530500

[B44] Palusa S. G. Ali G. S. Reddy A. S. (2007). Alternative splicing of pre-mRNAs of Arabidopsis serine/arginine-rich proteins: regulation by hormones and stresses. Plant J. 49, 1091–1107. 10.1111/j.1365-313X.2006.03020.x 17319848

[B45] Papp I. Mur L. A. Dalmadi A. Dulai S. Koncz C. (2004). A mutation in the cap binding protein 20 gene confers drought tolerance to Arabidopsis. Plant Mol. Biol. 55, 679–686. 10.1007/s11103-004-1680-2 15604709

[B46] Reichel M. Liao Y. Rettel M. Ragan C. Evers M. Alleaume A. M.. (2016). In planta determination of the mRNA-binding proteome of Arabidopsis etiolated seedlings. Plant Cell 28, 2435–2452. 10.1105/tpc.16.00562 27729395 PMC5134986

[B47] Smith T. F. Gaitatzes C. Saxena K. Neer E. J. (1999). The WD repeat: a common architecture for diverse functions. Trends Biochem. Sci. 24, 181–185. 10.1016/S0968-0004(99)01384-5 10322433

[B48] Sonnhammer E. L. Ostlund G. (2015). InParanoid 8: orthology analysis between 273 proteomes, mostly eukaryotic. Nucleic Acids Res. 43, D234–D239. 10.1093/nar/gku1203 25429972 PMC4383983

[B49] Sorenson R. Bailey-Serres J. (2014). Selective mRNA sequestration by OLIGOURIDYLATE-BINDING PROTEIN 1 contributes to translational control during hypoxia in Arabidopsis. Proc. Natl. Acad. Sci. U.S.A. 111, 2373–2378. 10.1073/pnas.1314851111 24469793 PMC3926019

[B50] Sysoev V. O. Fischer B. Frese C. K. Gupta I. Krijgsveld J. Hentze M. W.. (2016). Global changes of the RNA-bound proteome during the maternal-to-zygotic transition in Drosophila. Nat. Commun. 7:12128. 10.1038/ncomms12128 27378189 PMC4935972

[B51] Tanabe N. Yoshimura K. Kimura A. Yabuta Y. Shigeoka S. (2007). Differential expression of alternatively spliced mRNAs of Arabidopsis SR protein homologs, atSR30 and atSR45a, in response to environmental stress. Plant Cell Physiol. 48, 1036–1049. 10.1093/pcp/pcm069 17556373

[B52] Thandapani P. O'Connor T. R. Bailey T. L. Richard S. (2013). Defining the RGG/RG motif. Mol. Cell 50, 613–623. 10.1016/j.molcel.2013.05.021 23746349

[B53] Vacic V. Uversky V. N. Dunker A. K. Lonardi S. (2007). Composition profiler: a tool for discovery and visualization of amino acid composition differences. BMC Bioinform. 8:211. 10.1186/1471-2105-8-211 17578581 PMC1914087

[B54] Van Dyke M. W. Nelson L. D. Weilbaecher R. G. Mehta D. V. (2004). Stm1p, a G4 quadruplex and purine motif triplex nucleic acid-binding protein, interacts with ribosomes and subtelomeric Y' DNA in *Saccharomyces cerevisiae*. J. Biol. Chem. 279, 24323–24333. 10.1074/jbc.M401981200 15044472

[B55] Weber C. Nover L. Fauth M. (2008). Plant stress granules and mRNA processing bodies are distinct from heat stress granules. Plant J. 56, 517–530. 10.1111/j.1365-313X.2008.03623.x 18643965

[B56] Zhou Z. Licklider L. J. Gygi S. P. Reed R. (2002). Comprehensive proteomic analysis of the human spliceosome. Nature 419, 182–185. 10.1038/nature01031 12226669

